# Development of a Multi-Criteria Decision Analysis Rating Tool to Prioritize Real-World Evidence Questions for the Canadian Real-World Evidence for Value of Cancer Drugs (CanREValue) Collaboration

**DOI:** 10.3390/curroncol30040286

**Published:** 2023-03-28

**Authors:** Ambica Parmar, Wei Fang Dai, Francois Dionne, Marc Geirnaert, Avram Denburg, Tarry Ahuja, Jaclyn Beca, Sylvie Bouchard, Carole Chambers, Melissa J. Hunt, Don Husereau, Elena Lungu, Valerie McDonald, Rebecca E. Mercer, Gunita Mitera, Caroline Muñoz, Rohini Naipaul, Stuart Peacock, Tanya Potashnik, Mina Tadrous, Pam Takhar, Marianne Taylor, Maureen Trudeau, Danica Wasney, Scott Gavura, Kelvin K. W. Chan

**Affiliations:** 1 Division of Medical Oncology & Hematology, Department of Medicine, Sunnybrook Health Sciences Centre, Toronto, ON M4N 3M3, Canada; 2 Temerty Faculty of Medicine, University of Toronto, Toronto, ON M5S 1A8, Canada; 3 Prioritize Consulting Ltd., Vancouver, BC V6S 2B3, Canada; 4 CancerCare Manitoba, Winnipeg, MB R3E 0V9, Canada; 5 Division of Haematology/Oncology, The Hospital for Sick Children, Toronto, ON M5G 1X8, Canada; 6 Canadian Agency for Drugs and Technologies in Health, Ottawa, ON K1S 5S8, Canada; 7 Ontario Health (Cancer Care Ontario), Toronto, ON M5G 2L3, Canada; 8 Canadian Centre for Applied Research in Cancer Control, Toronto, ON M5G 2L3, Canada; 9 Institut National d’Excellence en Santé et en Services Sociaux (INESSS), Québec, QC G1V 4M3, Canada; 10 Alberta Health Services, Calgary, AB T5J 3E4, Canada; 11 Health Canada, Ottawa, ON K1A 0K9, Canada; 12 School of Epidemiology and Public Health, University of Ottawa, Ottawa, ON K1G 5Z3, Canada; 13 Patented Medicine Prices Review Board, Ottawa, ON K1P 1C1, Canada; 14 Independent Researcher, Toronto, ON M6G 2V3, Canada; 15 Canadian Association of Provincial Cancer Agencies, Toronto, ON M5H 1J8, Canada; 16 BC Cancer Agency, Vancouver, BC V5Z 1G1, Canada; 17 Leslie Dan Faculty of Pharmacy, University of Toronto, Toronto, ON M5S 3M2, Canada; 18 BC Cancer, Kelowna, BC V1Y 5L3, Canada

**Keywords:** oncology, decision-analysis, health technology assessment, multi-criteria decision analysis

## Abstract

The Canadian Real-world Evidence for Value of Cancer Drugs (CanREValue) collaboration developed an MCDA rating tool to assess and prioritize potential post-market real-world evidence (RWE) questions/uncertainties emerging from public drug funding decisions in Canada. In collaboration with a group of multidisciplinary stakeholders from across Canada, the rating tool was developed following a three-step process: (1) selection of criteria to assess the importance and feasibility of an RWE question; (2) development of rating scales, application of weights and calculating aggregate scores; and (3) validation testing. An initial MCDA rating tool was developed, composed of seven criteria, divided into two groups. Group A criteria assess the importance of an RWE question by examining the (1) drug’s perceived clinical benefit, (2) magnitude of uncertainty identified, and (3) relevance of the uncertainty to decision-makers. Group B criteria assess the feasibility of conducting an RWE analysis including the (1) feasibility of identifying a comparator, (2) ability to identify cases, (3) availability of comprehensive data, and (4) availability of necessary expertise and methodology. Future directions include partnering with the Canadian Agency for Drugs and Technology in Health’s Provincial Advisory Group for further tool refinement and to gain insight into incorporating the tool into drug funding deliberations.

## 1. Introduction

The rapid pace of cancer drug development over the past decade has led to new challenges for health care spending, particularly in publicly funded health care systems [1]. In Canada, payers are increasingly required to make cancer drug funding decisions based on early clinical data that may be non-comparative or that lack mature survival outcomes. This means economic evaluations that characterize value-for-money require statistical extrapolation and mathematical modelling which contribute to significant uncertainty about long-term and comparative clinical benefit and value [2]. The generation of real-world evidence (RWE) after a cancer drug is launched can confirm whether policy-makers and payers are obtaining the clinical outcomes and value-for-money they expect. 

The Canadian Real-world Evidence for Value in Cancer Drugs (CanREValue) Collaboration was created to develop a framework to generate and use RWE to support cancer drug funding decisions [3]. CanREValue’s framework provides a process for evidence-based reassessment of cancer drug funding recommendations by health technology assessment (HTA) organizations. CanREValue established the Planning and Drug Selection Working Group (WG) to identify systemic therapies with identified uncertainties (e.g., lack of mature survival data, lack or inappropriate use of a comparator, use of surrogate endpoints) at the time of initial drug funding that may be resolved through the generation of RWE. WG members include relevant stakeholders integral to cancer drug funding decisions in Canada including regulatory agencies (e.g., Health Canada, PMPRB), HTA agencies, public payers, clinicians, patient representatives, pricing negotiation bodies, and researchers. 

Given the potentially large number of uncertainties that may be addressed through RWE analyses, there is a need to assess and prioritize RWE questions that are relevant and feasible for public payers when making drug-funding decisions. Multi-criteria decision analysis (MCDA) is an approach that can be used to support complex decision-making by allowing the assessment of multiple different viewpoints across a broad range of stakeholders [4]. Through a structured approach, MCDA can facilitate transparency in decision-making processes and improve the quality and consistency of decisions [5]. MCDA use in health care is increasing given its utility as a decision-aid in complex decision-making [4,5,6]. For example, MCDA has been used in health policy to support HTAs for regulatory or reimbursement decisions [7,8,9], as well as in clinical decision-making at the patient-level [10,11] and has been adopted by many international HTA agencies (e.g., IQWiG (Germany), INESSS (Quebec, QC, Canada)) [12,13]. MCDA was chosen by the CanREValue Planning and Drug Selection WG as a method to assess and prioritize potential RWE questions that address uncertainties in cancer drug funding recommendations with the intent to inform national HTA agencies and public payers. In this article, we describe the process undertaken by the WG to develop the MCDA rating tool.

## 2. Development of an MCDA Rating Tool

The development of the MCDA rating tool was led by an expert in MCDA application (FD) and developed in collaboration with the Planning and Drug Selection WG. The methods used to inform our development process have been applied successfully in numerous health care settings in Canada and are in accordance with the International Society of Pharmacoeconomics and Outcomes Research (ISPOR) Good Practices Guidelines [12,14]. On the basis of those recommendations, the rating tool was developed in a stepwise approach: (1) selection of criteria to assess the importance and feasibility of an RWE question; (2) development of rating scales, application of weights to each criterion, and calculating aggregate scores and (3) validation testing of the MCDA rating tool and making adjustments, as necessary. The development of the MCDA rating tool was an iterative process and WG members had multiple opportunities to provide input throughout the development process to ensure the tool is helpful to both decision-makers and end-users.

### 2.1. Selection of Criteria to Assess the Importance and Feasibility of an RWE Question

In consultation with an expert in MCDA development (FD), a set of draft criteria to assess and prioritize potential RWE questions was conceptualized. This set included 10 criteria categorized into two groups. Group A assessed the importance of the proposed RWE question or uncertainty identified during assessment of drug funding recommendations and Group B assessed the likelihood of finding an answer to the RWE question or resolving the identified uncertainty (Appendix A). As recommended by ISPOR, feedback on this initial set of criteria was elicited from the multi-disciplinary WG to ensure criteria were complete, operational to end-users, and sufficiently independent from each other to avoid redundancy and overlap [12]. As a result of this step, several modifications were made including the removal of select criteria, clarifying instructions and rating descriptions associated with each criteria. For instance, elicited feedback highlighted the importance of considering reasonable time frames when assessing sample size of the target and comparator population. With the integration of this factor into the criteria of “Sample Size” and “Comparator” the original “Time” criterion was removed. Through this iterative process, the WG was able to reach consensus on the modified list of criteria, allowing the subsequent development of performance measures, rating scales and weighting (Table 1).

Next, the WG developed performance measures unique to each criterion. The performance measures encompass either quantitative or qualitative metrics to assess the RWE question. For instance, assessment of the drug’s perceived incremental benefit is quantitative based upon reported clinical outcomes (e.g., overall survival, progression-free survival, response rate) of the therapy of interest as observed in either clinical trial evidence or through indirect comparisons. Alternatively, a qualitative assessment by expert opinion is required to assess the relevance of the proposed RWE question (Table 2).

### 2.2. Developing Rating Scales, Application of Weights for Each Criterion and Calculating Aggregate Scores

Rating scales allow for scoring of the developed performance measures [5]. A unique rating scale was developed to score each criterion on a 3-point scale with a score of 3 depicting high performance of the proposed RWE study on a given criterion. A 3-point scale was chosen to reduce cognitive burden and maximize efficiency among the end-users while meeting the objective of the MCDA rating tool to support prioritization (i.e., ranking) of potential RWE questions (Table 2) [5]. 

Weights were developed for each criterion in the MCDA rating tool to incorporate the relative importance of each criterion in the assessment of a proposed RWE study. The process of weighting the criteria involved multi-disciplinary stakeholder engagement to ensure the preferences of relevant end-users were elicited and incorporated [5]. Note that there is no “correct” set of weights for any list of criteria. The appropriate weights are those that reflect the values of the organization that will use the criteria (i.e., they are context dependent). The first step in developing the weights for each criterion was to survey all members of the Planning and Drug Selection WG individually as to the three criteria that they felt were the most important and the three criteria that they felt were least important, but still relevant. From the 13 WG members, a total of eight responses from key representative stakeholders were obtained, revealing the top-rated most important criteria to be: (1) relevance of uncertainty; (2) drug’s perceived incremental benefit; (3) impact of uncertainty and (4) data. The least important criteria were: (1) expertise; (2) methodology; (3) magnitude of uncertainty. Of note, while not all WG members participated in the survey, the responses encompassed sufficient perspectives from the range of different stakeholders (i.e., some organizations had multiple WG members and only one response was recorded). The list of top-rated most and least important criteria was used to develop a set of weights for the WG to discuss by the MCDA expert (FD). Each criterion began with an equal weight (e.g., with 10 criteria, the starting point is a weight of 10% each). Responses received in the aforementioned survey was then used to increase, decrease, or leave unchanged the weight of each criterion. For example, the top-rated “most-important” criterion had the largest weight increase. As part of the final step in developing the weights, WG members discussed the proposed weights and identified those requiring additional adjustments. Final adjustments to the proposed weights were applied following this discussion (Table 2).

Consistent with other applications of MCDA in health care, an additive model is used to calculate aggregate scores and thus was adopted for our rating tool [5]. Application of the weights to a user’s rating for each criterion will generate an aggregate weighted score for a proposed RWE study. This weighted score is an estimate of the value of a proposed RWE study and can then be used to guide deliberation to prioritize high-value RWE studies.

### 2.3. Validation Testing of the MCDA Tool

Following development of the initial MCDA rating tool (including criteria, rating scales and weights), two pilot tests were performed in June and December 2020 to evaluate the usability of the tool in assessing and prioritizing proposed RWE projects. Each pilot test was conducted with a group of multi-disciplinary participants made up of clinical experts, methodologists, and various health policy experts. During the pilot tests, five participants were provided a mock RWE study proposal and were asked to individually rate the proposal using the MCDA rating tool. The two mock RWE proposals were: (a) “What is the real-world comparative effectiveness of first-line crizotinib, as compared to platinum-based chemotherapy for patients with metastatic non-small cell lung cancer that harbors a *ROS-1* rearrangement?”, and (b) “What is the real-world comparative effectiveness of nivolumab in patients with classical Hodgkin’s Lymphoma with evidence of disease progression following autologous stem cell transplantation and brentuximab vedotin, as compared to standard single-agent chemotherapy or pembrolizumab immunotherapy?” Following each pilot test, qualitative feedback was elicited from participants through the use of surveys and roundtable discussions, to assess the overall usability of the MCDA rating tool and its applicability in assessing the value of potential RWE projects. 

Through this process, participants consistently reported that the MCDA rating tool was easy to use. Participants felt that the MCDA rating criteria assessed all relevant attributes needed to understand the importance and feasibility of RWE proposals. Consensus on criterion rating was consistently achieved through discussion. Instances of initial discordant results often occurred when users reported uncertainty in their rating of specific criteria, most commonly with criterion 1 (drug’s perceived clinical benefit), criterion 4 (relevance of uncertainty), criterion 7 (data) and criterion 9 (methodology). However, by engaging a multi-disciplinary committee of experts, members were able to share pertinent expertise and unique perspectives to address each criterion in the MCDA rating tool which contributed to meaningful discussion and helped to achieve consensus. There was agreement that future committees should continue to include clinical experts, methodologists and decision-makers involved in Canadian drug funding decisions. We also identified several other critical stakeholders to be included in future iterations, such as a bioethicists and patient representatives. Additionally, many users also noted overlap in their interpretation of criterion 3 (impact of uncertainty) and criterion 4 (relevance of uncertainty). Through careful consideration of this feedback, it was felt that criterion 3 was assessing a similar attribute as criterion 4. Therefore, to avoid redundancy in the rating tool, criterion 3 was removed. Similarly, many users noted overlap in their assessment and rating of criterion 8 (expertise) and criterion 9 (methodology) prompting merging of these two criteria to avoid redundancy. The modified MCDA rating tool inclusive of seven criteria can be found in Table 2. 

## 3. Summary and Future Directions

Through a stepwise, iterative process, CanREValue’s Planning and Drug Selection WG has created and validated a MCDA rating tool that can be used to assess the value and support the prioritization of proposed RWE studies intended to reduce uncertainties in Canadian cancer drug funding recommendations. As the number of cancer drugs in the public drug funding pipeline continues to grow, use of the MCDA rating tool is expected to support a process that identifies the most pertinent and relevant post-market RWE studies. 

A notable limitation of the current MCDA rating tool is the reliance on expert opinions to measure performance for many of the criteria. To mitigate this, as our validation testing shows, effective use of the MCDA rating tool into decision-making for utilization of RWE in drug funding recommendations will require multi-disciplinary committees, with all relevant clinical, health policy and methodology experts included, to adequately apply the MCDA rating tool and minimize any potential uncertainty with generated scores. Additionally, the quantitative measures used to evaluate the magnitude of uncertainty in survival estimates and/or estimates of cost-effectiveness (as interpreted through reported incremental cost-effectiveness ratios) may be imprecise given their derivation from consensus opinion among the WG (Table 2). However, these quantitative measures were included to serve as a guide for interpretation of the performance measure. In our validation testing, we did not observe any major discordances with application of this criterion among different users. 

Future directions for the CanREValue’s Planning and Drug Selection WG include ongoing engagement with stakeholders in the cancer drug funding pathway to identify opportunities for application of the MCDA rating tool and to discuss implementation strategies. This includes a recent partnership with the Canadian Agency for Drugs and Technology in Health (CADTH) Provincial Advisory Group (PAG) to gain important insight into how this tool could be used to identify potential RWE studies during initial drug funding deliberations, as well, to promote ongoing discussions of how planned RWE studies may be used to support initial drug funding recommendations.

## Figures and Tables

**Table 1:** Updated MCDA Rating Tool Criteria.

Criteria
**Group A—Criteria to Assess the Importance of the RWE Question**
Drug’s perceived incremental benefit: Extent of perceived net clinical benefit of the therapy compared to existing options based upon clinical evidence (accounting for quality of evidence, unmet patient need, and any other contextual factors)
Magnitude of uncertainty: Magnitude of the uncertainty identified in cancer drug funding deliberations (the uncertainty can be about toxicity, clinical effectiveness, quality-of-life, treatment pattern, generalizability of benefits, costs, etc.)
Impact of uncertainty: Potential impact of the uncertainty on the total incremental benefits and/or total incremental costs (balance between incremental benefits and incremental costs) compared to relevant Canadian comparator treatment
Relevance of uncertainty: Relevance to decision-makers (for example, consider the potential effect of the identified uncertainty on funding status, funding pathways, budget-impact, etc.)
**Group B—Criteria to Assess the Likelihood of Finding an Answer to the RWE question**
Comparator: Likelihood that a relevant Canadian comparator population of sufficient sample size can be identified within a reasonable time frame (i.e., within time to be relevant to the funding decision)
Sample size: Extent to which it is likely that there will be enough patients to have a sufficient sample size within a reasonable time frame (i.e., within time to be relevant to the funding decision)
Data: Likelihood that there will be available, high quality and complete data for the cohort receiving the therapy of interest and the comparator, including data for important patient and clinical characteristics to ensure comparability between groups, as well as relevant outcomes
Expertise: Availability of expertise to conduct the RWE analysis
Methodology: Availability of appropriate methodology (with consideration given to current data availability and the clinical context)

Abbreviations: ICER: incremental cost-effectiveness ratio; pCODR: pan-Canadian Oncology Drug Review; RWE: real-world evidence.

**Table 2:** Current MCDA Rating Tool.

Criteria	Rating Scale			Weight
	1	2	3	
Group A—Criteria to Assess the Importance of the RWE Question				
Drug’s perceived incremental benefit: The objective of this criterion is to evaluate the perceived clinical benefit of the therapy compared to existing options. The ‘perceived’ clinical benefit is based on the currently available objective evidence (including primary clinical trial data and indirect comparisons ^1^) and expected long-term outcomes (in the setting of immature data).	Rated using the European Society of Medical Oncology (ESMO) Magnitude of Clinical Benefit Scale (MCBS) v1.0. [15]			17.65
	**Minimal to low clinically meaningful incremental benefit**, as evidenced by: (a) overall survival benefit demonstrated through a hazard ratio > 0.65 and/or gain in median overall survival of <2.5 months, as compared to current standard(s) of care, for therapies with a median overall survival of <1 year; or (b) overall survival benefit demonstrated through a hazard ratio of >0.70 and/or gain in median overall survival of <3 months, as compared to current standard(s) of care, for therapies with a median overall survival > 1 year; or (c) median progression free survival benefit demonstrated through a hazard ratio of > 0.65, as compared to current standard(s) of care; or) clinical benefit demonstrated in an alternative outcome (including improvements in response rate, quality-of-life or other clinically meaningful outcome).	**Moderate clinically meaningful incremental benefit**, as evidenced by: (a) survival benefit demonstrated by a hazard ratio < 0.65 and gain in median survival of 2.5–2.9 months, as compared to current standard(s) of care, for therapies with median overall survival < 1 year; or (b) overall survival benefit demonstrated by a hazard ratio < 0.70 and gain in median overall survival of 3-4.9 months, as compared to current standard(s) of care, for therapies with median overall survival > 1 year; or (c) progression-free survival benefit demonstrated by a hazard ratio < 0.65 and gain > 1.5 months, as compared to current standard(s) of care.	**Substantial clinically meaningful incremental benefit**, as evidenced by: (a) overall survival benefit demonstrated through a hazard ratio of <0.65 and gain in median overall survival of 2.5–3.0 months, as compared to current standard(s) of care, for therapies with median overall survival < 1 year; or (b) overall survival benefit demonstrated through a hazard ratio < 0.70 and gain in median overall survival of >5 months, as compared to current standard(s) of care, for therapies with median overall survival >1 year.	
Magnitude of uncertainty: The objective of this criterion is to assess the degree of uncertainty in question (the uncertainty can be about toxicity, clinical effectiveness, quality-of-life, treatment sequence, generalizability of benefits, costs or other).	**Minimal uncertainty**: This can be based upon either a qualitative assessment or quantitative assessment (the latter can be conceptualized as a <10% variation in either of the following: (a) the confidence intervals around the survival estimates; (b) the upper and lower range of ICERs from the pCODR assessment ^1^).	**Moderate uncertainty**: This can be based upon either a qualitative assessment or quantitative assessment (the latter can be conceptualized as a 10–25% variation in either of the following: (a) the confidence intervals around the survival estimates; (b) the upper and lower range of ICERs from the pCODR assessment ^1^).	**Substantial uncertainty**: This can be based upon either a qualitative assessment or quantitative assessment (the latter can be conceptualized as a >25% variation in either of the following: (a) the confidence intervals around the survival estimates; (b) the upper and lower range of ICERs from the pCODR assessment ^1^).	10.6
Relevance of uncertainty: The objective of this criterion is to assess the relevance of resolving the uncertainty to decision-makers (i.e., what is the likelihood that resolving the uncertainty with new evidence will alter the funding status or clinical treatment recommendations).	**Indirect relevance**: As assessed by expert opinions, there is an expected low likelihood for new evidence to facilitate a change in funding status (i.e., facilitate drug price re-negotiations) and/or change in clinical treatment recommendations (i.e., indicated patient populations or treatment sequence).	**Moderate relevance**: As assessed by expert opinions, there is uncertainty in the likelihood for new evidence to facilitate a change in funding status (i.e., facilitate drug price re-negotiations) and/or change in clinical treatment recommendations (i.e., indicated patient populations or treatment sequence).	**Substantial relevance**: As assessed by expert opinions, there is an expected high likelihood for new evidence to facilitate a change in funding status (i.e., facilitate drug price re-negotiations) and/or change in clinical treatment recommendations (i.e., indicated patient populations or treatment sequence).	18.8
Group B—Criteria to Assess the Feasibility of the RWE Project				
Comparator: The objective of this criterion is to assess the likelihood that a relevant historical or contemporaneous Canadian comparator population, of sufficient size, can be identified within a reasonable time frame (i.e., within time to be relevant to the funding decision, for contemporaneous control). A ‘relevant’ comparator is a group that has been treated according to current Canadian standard of care regimen.	**Substantial concern**: Unlikely to identify an appropriate comparator population within a reasonable time due to absence of clear standard-of-care therapy (i.e., >2 relevant standard-of-care treatments currently available or evolving standard-of-care treatment) and/or low-volume patient population.	**Moderate concern**: Moderate concern for the identification of an appropriate comparator population due to absence of clear standard-of-care therapy (i.e., 2 relevant standard-of-care treatments currently available) and/or moderate-volume patient population.	**Low concern**: Appropriate comparator population will be easily identified due to a well-defined standard of care therapy and high-volume patient population.	11.8
Cases: The objective of this criterion is to assess the likelihood that there will be enough patients receiving the treatment in question to have a sufficient sample size within a reasonable time frame (i.e., within time to be relevant to the funding decision).	**Substantial concern**: Unlikely to establish a sufficient sample size (with appropriate follow-up for relevant outcome(s)) within a reasonable time ^2^ based upon expected incidence of disease (using Canadian provincial estimates) and required sample size for analysis.	**Moderate concern**: Likely to establish a sufficient sample size, based upon expected incidence of disease (using Canadian provincial estimates) but unlikely to have follow-up for relevant outcome(s) within a reasonable time ^2^ based upon expected incidence (using Canadian provincial estimates) and required sample size for analysis.	**Low concern**: Very likely to establish a sufficient sample size (with appropriate follow-up for relevant outcome(s)) within a reasonable time ^2^ based upon expected incidence of disease (using Canadian provincial estimates) and required sample size for analysis.	14.1
Data: The objective of this criterion is to assess the quality of data available in at least one Canadian province to address the uncertainty. This requires an assessment of the availability and completeness of data for both the exposed and comparator cohorts pertaining to: (a) data for relevant patient and disease characteristics to account for important co-variates, ensure un-biased comparability between groups and measure relevant outcomes +/− (b) data for relevant costing inclusive of total health care costs accrued during treatment (ex. systemic treatment, planned and unplanned health care resource utilization).	**Substantial concern**: Substantial concern for the availability of high-quality and complete data for both exposed and comparator cohorts in known real-world databases (as assessed by an absence of ≥1 of the following: (a) patient and/or disease characteristics required to define current funding eligibility; (b) >2 relevant patient and/or disease co-variates; (c) ability to identify primary systemic treatment, inclusive of line-of-therapy).	**Moderate concern**: Moderate concern for the availability of high-quality and complete data for both exposed and comparator cohorts in known real-world databases (as assessed by an absence of ≥1 of the following: (a) 1–2 relevant patient and/or disease co-variates; (b) ability to identify prior or subsequent treatment inclusive of line-of-therapy).	**Low concern**: No expected issues in accessing high-quality and complete data in known real-world databases.	17.65
Expertise and Methodology: The objective of this criterion is to evaluate the availability of required expertise (ex. clinical experts, data analysts and methodologists) and methodology to conduct the study.	**Substantial concern**: Expected challenges to find the necessary expertise and need to develop new methods to conduct the study, with above limitations in data taken into consideration (if applicable).	**Moderate concern**: Expected challenges to find the necessary expertise or need to develop new methods to conduct the study, with above limitation in data taken into consideration (if applicable).	**Low concern**: No expected issues with the availability of the necessary expertise and no new methods required to conduct the study.	9.4

^1^ Please refer to the pCODR economic guidance report for details on pCODR’s reanalysed ICERs. ^2^ Reasonable time: time required is less than half the time of remaining patency duration. Abbreviations: ICER: incremental cost-effectiveness ratio; pCODR: pan-Canadian Oncology Drug Review.

## Data Availability

Not applicable.

## Appendix A

**Table A1:** Initial set of MCDA criteria.

Criteria
Group A—Criteria to Assess the Importance of the RWE Question or Uncertainty Identified
Drug’s perceived incremental benefit: Extent of perceived net clinical benefit of the therapy compared to existing options (accounting for quality of evidence, unmet need, and any other contextual factors). The ‘perceived’ net clinical benefit is based on the currently available evidence.
Magnitude of uncertainty identified: Magnitude of the uncertainty identified in cancer drug funding deliberations (the uncertainty can be about toxicity, clinical effectiveness, quality-of-life, treatment pattern, generalizability of benefits, costs, etc.)
Impact of uncertainty: Potential impact of the uncertainty on the balance between incremental benefits and incremental costs
Relevance to payer: Consider the potential effect of the identified uncertainty on funding status, funding pathways, budget-impact analysis, etc.
Group B—Criteria to Assess the Likelihood of Finding an Answer to the RWE question or Resolving the Identified Uncertainty
Sample size: Extent to which it is likely that there will be enough patients to have a sufficient sample size
Comparator: Likelihood that a relevant Canadian comparator population can be identified. A ‘relevant’ comparator is a group that has been treated according to current Canadian standard of care regimen.
Time: Likelihood that there is enough time to accrue and follow patients for the outcomes of interest
Data: Likelihood that there will be available and relatively complete data for cohort receiving therapy and the comparator, including data for important patient and clinical characteristics to ensure comparability between groups, as well as measure and relevant outcomes
Expertise: Availability of expertise to conduct the RWE analysis
Methodology: Availability of appropriate methodology (with consideration given to current data availability and the clinical context)
